# Relationship between body roundness index and obstructive sleep apnea: a population-based study

**DOI:** 10.3389/fnut.2025.1531006

**Published:** 2025-03-26

**Authors:** Haoyue Gao, Rundong Zhang, Peipei Wang, Dai Liu, Jianxing Han, Bei Wang

**Affiliations:** ^1^Department of Respiratory, The Second Hospital of Shanxi Medical University, Taiyuan, China; ^2^Department of Stomatology, The Second Hospital of Shanxi Medical University, Taiyuan, China

**Keywords:** obstructive sleep apnea, body roundness index, body mass index, central obesity, visceral fat accumulation

## Abstract

**Objectives:**

This study aimed to assess the relationship between body roundness index (BRI) and obstructive sleep apnea (OSA) risk and the difference in the ability of BRI and body mass index (BMI) to identify people at high risk for OSA in different conditions.

**Methods:**

This study was based on the National Health and Nutrition Examination Survey (NHANES) from 2005 to 2008 and 2015 to 2018. Participants were categorized into OSA high-risk and OSA low-risk groups *via* questionnaires about sleep. The potential relationship between BRI and high risk for OSA was explored using several statistical methods, including weighted logistic regression models, receiver operating characteristic curves (ROC), restricted cubic spline curves (RCS), interaction tests, and subgroup analyses.

**Results:**

A total of 9,495 participants were included in this study, including 3,155 in the high-risk group and 6,340 in the low-risk group. In the crude model, BRI was positively associated with a high risk for OSA (OR = 1.23; 95% CI: 1.20–1.27). After adjusting for all covariates, higher BRI quartiles (Q4) were positively associated with high risk for OSA (OR = 3.22; 95% CI: 2.57–4.04). The RCS demonstrated that BRI was non-linearly associated with OSA risk. ROC analyses showed that BRI was better at identifying those at high risk for OSA in the normal-weight population than BMI. Subgroup analyses revealed stronger correlations in non-hypertensive and non-smoking populations.

**Conclusions:**

There is a non-linear positive correlation between BRI and OSA risk, and early monitoring and managing BRI can help to identify people at high risk for OSA as early as possible and reduce the risk.

## 1 Introduction

Obstructive sleep apnea (OSA) is a common clinical sleep disorder caused by recurrent narrowing or collapse of the airway during sleep and characterized by snoring, apnea, and excessive daytime sleepiness (1, 2). The prevalence of moderate to severe OSA is estimated to be approximately 13% in men and 6% in women among adults aged 30–70 years in the United States (3). OSA can lead to a variety of complications, including hypertension, metabolic syndrome, stroke, and diabetes. In addition, the 5-year mortality rate for untreated OSA can be as high as 11%−13% (4).

It is now widely recognized that obesity is strongly linked to OSA (5). Obesity not only significantly raises the incidence and severity of OSA (6) but also increases the risk of cardiovascular diseases (CVD) among patients suffering from OSA (7). The official clinical practice guidelines of the American Thoracic Society indicate that weight loss intervention is an effective measure to improve the severity of OSA and cardiometabolic comorbidities (8). Body mass index (BMI) has long been the primary indicator for assessing obesity due to its convenient measurement (9). However, it has been shown that OSA is associated with abdominal fat accumulation rather than subcutaneous fat area (10). Therefore, the accuracy of BMI has been questioned because of its inability to differentiate between muscle and fat nor to assess fat distribution (11). Although waist circumference can be used as a reference for quantifying central obesity, it is unreliable because it cannot be adjusted for differences due to height. In order to more accurately assess fat distribution, Thomas et al. developed the Body Rounds Index (BRI), which assesses the roundness of the body through an elliptical model that reflects the shape of the human body (12). The BRI incorporates height and waist circumference variables into its calculation, which can help to more accurately assess the degree of central obesity or the accumulation of visceral adipose tissue. Numerous studies have shown a significant correlation between BRI levels and the incidence of cardiovascular disease, depression, metabolic syndrome, and type 2 diabetes (13–16).

Recent studies have shown that BRI is significantly associated with CVD risk in patients with OSA (17). Although two previous studies have evaluated the relationship between BRI and OSA (18, 19), the previous studies primarily treated BRI as one of multiple metrics and did not compare the specific differences between BRI and BMI in screening for those at high risk for OSA. Therefore, by analyzing data from the National Health and Nutrition Examination Survey (NHANES) database, this study aims to explore the potential relationship between BRI and OSA risk, investigate the differences in the ability of BRI and BMI to identify high-risk OSA populations under different circumstances, and observe the performance differences of BRI in different population subgroups. Early identification of people at high risk for OSA can help develop appropriate treatment plans to improve patient's quality of life and reduce complications.

## 2 Methods

### 2.1 Study population and design

The NHANES survey is a national cross-sectional survey of physical health and nutritional status conducted by the National Center for Health Statistics (NCHS). Data are collected biennially through a multi-stage, stratified sampling method, including demographic data, dietary information, physical examination data, laboratory test results, and questionnaires. The NHANES study was approved by the Research Ethics Review Board of the NCHS, and all participants signed a written informed consent form. Detailed information about NHANES can be obtained from the official website at http://www.cdc.gov/nchs/nhanes.

**Figure 1:**
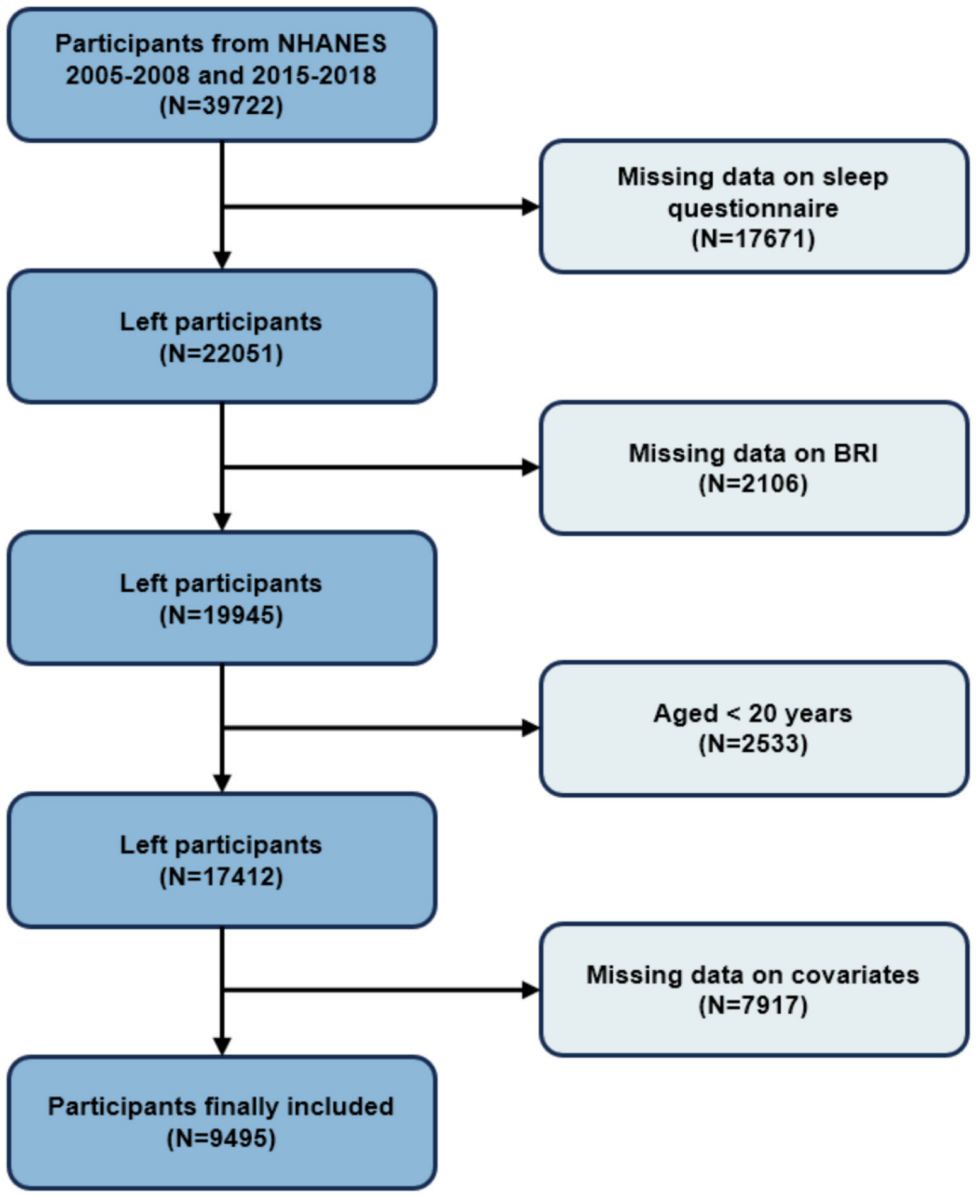
The flow chart of population selection.

The purpose of this study was to explore the relationship between BRI and the risk of OSA in adults aged 20 years or older in the United States. First, we determined the study period as 2005–2008 and 2015–2018 based on the period of questionnaires about sleep (20). During these periods, a total of 39,722 individuals were enrolled. Of these, 17,671 participants without completed sleep questionnaires were excluded. Additionally, individuals missing the BRI (2,106), younger than 20 years of age (2,533), and without a complete covariate (7,917) were excluded. As shown in Figure 1, there were 9,495 individuals included in this study.

### 2.2 High-risk for obstructive sleep apnea syndrome and its symptoms

Referring to previous studies, participants who answered “yes” to any of the following three questions were categorized as high-risk for OSA (21, 22). Conversely, participants who did not meet the criteria were considered low-risk for OSA.

(1) “In the past 12 months, did you snore 3 or more nights a week while you were sleeping?”

(2) “In the past 12 months, did you snort, gasp, or stop breathing 3 or more nights a week while you were asleep?”

(3) “In the past month, did you feel excessively or overly sleepy 16-30 times a month during the day?”

### 2.3 Body roundness index

The BRI is a new index for assessing central obesity and visceral fat accumulation, calculated from height (cm) and waist circumference (cm) (12). To ensure accuracy, the standing height and waist circumference of the participants were measured by professional health technicians at a mobile examination center. In this study, the BRI was divided into 4 groups by quartiles to investigate its correlation with the risk of OSA. The BRI calculation formula is as follows:


$$BRI=364.2-365.5\times \sqrt{1-\frac{{\left(\frac{WC}{2\pi }\right)}^{2}}{{\left(0.5Height\right)}^{2}}}\;$$

### 2.4 Other covariates

Appropriate covariates were selected based on previous researches, including age, sex, race and ethnicity, marital status, poverty-income ratio (PIR), educational level, smoking status, drinking status, CVD, hypertension, diabetes mellitus (DM), triglycerides (TG), total cholesterol (TC), and high-density lipoprotein (HDL). Questionnaire information was collected by professional staff using standardized questionnaires during home interviews. Smoking status was classified according to whether the participant had ever smoked 100 or more cigarettes. Drinking status was defined as consuming 2 (female)/3 (male) drinks or more per day. Criteria for the diagnosis of diabetes include (1) self-reported diagnosis by a physician, (2) HbA1c 6.5 % or fasting blood glucose ≥7.0 mmol/L, (3) random blood glucose ≥11.1 mmol/L, (4) 2-h plasma glucose after oral glucose tolerance test ≥11.1 mmol/L, and (5) use of diabetes medications or insulin. Diagnostic criteria for hypertension include (1) self-reported diagnosis by a physician, (2) taking antihypertensive drugs, and (3) having a systolic blood pressure >140 mmHg and a diastolic blood pressure >90 mmHg. Cardiovascular diseases include coronary artery disease, heart failure, heart attack, stroke, and angina. BMI was calculated based on weight (kg) and height (m). BMI groups were defined as normal (BMI < 25 kg/m^2^), overweight (BMI 25 to < 30 kg/m^2^) and obese (BMI ≥ 30 kg/m^2^). TG, TC, and HDL levels were measured from blood samples. Detailed data collection procedures and measurements for all variables are available through the NHANES website (http://www.cdc.gov/nchs/nhanes).

### 2.5 Statistical analyses

Because NHANES is a complex multi-stage probability sampling study, weights were recalculated after combining the 2005–2008 and 2015–2018 datasets (WTMEC2YR/4). The BRI was categorized into quartiles (Q1 to Q4) from low to high in descriptive analyses. Categorical variables are shown as counts (N) and weighted percentages (%), while continuous variables are reported as means and standard deviations (SD). Weighted *t*-tests were used for continuous variables, and weighted chi-square tests were used for categorical variables to assess differences between low-risk and high-risk groups. To assess the diagnostic performance of the BRI for OSA risk and related symptoms, receiver operating characteristic curves (ROC) were plotted, and the area under the curves (AUC) was calculated. Multivariate logistic regression models were utilized to examine the odds ratios (OR) and 95% confidence intervals (95% CI) for the correlation between BRI and OSA risk. Variables with *P* < 0.1 in the univariate regression analysis were stepwise included in the adjusted model 1 and 2 (Supplementary Table 1). The crude model was not adjusted for any variables; model 1 was adjusted only for age, sex, marital status, PIR, and educational level; model 2 was adjusted for age, sex, marital status, PIR, educational level, DM, Hypertension CVD, TG, TC, HDL, and smoking status; and model 3 adjusted for age, sex, race, marital status, PIR, educational level, DM, Hypertension, CVD, TG, TC, HDL, smoking status, and drinking status. The generalized variance inflation factor (GVIF) in the regression models was < 2, indicating no high degree of multicollinearity among the covariates (Supplementary Table 2). Trends analyses were used to explore trends between BRI groups. Restricted cubic spline curves (RCS) were used to explore the non-linear association of BRI with OSA. Subgroup analyses and interaction tests were performed in Model 3 stratified by age, sex, race, marital status, PIR, education level, DM, hypertension, CVD, smoking status, and drinking status to investigate possible differences between populations. The study was statistically analyzed using R software (version 4.4.1), and *P* < 0.05 was considered statistically significant.

## 3 Results

### 3.1 Baseline characteristics

A total of 9,495 participants were included in this analysis, representing 119.5 million United States residents. Based on the risk of OSA, all participants were categorized into a low-risk group (*N* = 6,340) and a high-risk group (*N* = 3,155), and the weighted baseline characteristics of the participants are shown in Table 1. The mean age of the participants was 45.18 (0.39) years, and 51.95% were male. Compared to the low-risk group, participants in the high-risk group were more likely to be male, high school or general educational development (GED)/less than high school, married/living with a partner, smoking, older, and had higher TG, BMI, and BRI but lower HDL (*P* < 0.05). Similarly, these participants had a higher likelihood of comorbid DM, hypertension, and CVD (*P* < 0.05). As shown in Supplementary Table 3, the higher quartiles of the BRI had older participants, had a higher proportion of males, high school or GED/less than high school, married/living with a partner; higher TG, TC, and BMI; a higher prevalence of smoking, hypertension, DM, CVD; and a higher risk of OSA, snore, stop breathing and daytime sleepiness, but lower rates of TC and drinking (*P* < 0.05).

**Table 1:** Weighted baseline characteristics of participants.

**Characteristics**	**Total (*N =* 9,495)**	**Low-risk (*N =* 6,340)**	**High-risk (*N =* 3,155)**	***P*-value**
**Continuous variables, mean (SD)**				
Age (years)	45.18 (0.39)	44.39 (0.41)	46.82 (0.48)	< 0.0001
PIR	3.30 (0.04)	3.33 (0.04)	3.23 (0.06)	0.07
TG (mmol/L)	1.71 (0.03)	1.61 (0.03)	1.93 (0.05)	< 0.0001
TC (mmol/L)	5.03 (0.02)	5.01 (0.02)	5.07 (0.03)	0.1
HDL (mmol/L)	1.41 (0.01)	1.46 (0.01)	1.32 (0.01)	< 0.0001
BMI (kg/m^2^)	28.90 (0.13)	27.83 (0.16)	31.12 (0.18)	< 0.0001
BRI	5.23 (0.05)	4.87 (0.05)	5.97 (0.06)	< 0.0001
**Categorical variables**, ***n*** **(%)**				
**BRI quartile**				< 0.0001
Q1	2,373 (27.68)	1,927 (33.56)	446 (15.50)	
Q2	2,372 (25.22)	1,660 (26.23)	712 (23.13)	
Q3	2,375 (23.64)	1,521 (21.86)	854 (27.31)	
Q4	2,375 (23.46)	1,232 (18.35)	1,143 (34.05)	
**Sex**				< 0.0001
Female	4,319 (48.05)	3,016 (51.16)	1,303 (41.59)	
Male	5,176 (51.95)	3,324 (48.84)	1,852 (58.41)	
**Educational level**				0.01
Above high school	5,560 (65.91)	3,741 (67.25)	1,819 (63.14)	
High school or GED	2,173 (22.99)	1,414 (22.08)	759 (24.87)	
Less than high school	1,762 (11.10)	1,185 (10.67)	577 (11.99)	
**Race**				0.73
Non-Hispanic Black	1,856 (9.08)	1,218 (8.83)	638 (9.60)	
Mexican American	1,519 (7.80)	1,019 (7.79)	500 (7.82)	
Other Hispanic	845 (4.78)	562 (4.78)	283 (4.78)	
Other Race	916 (6.45)	616 (6.55)	300 (6.26)	
Non-Hispanic White	4,359 (71.89)	2,925 (72.05)	1,434 (71.54)	
**Marital status**				< 0.001
Living alone	3,389 (32.23)	2,351 (33.88)	1,038 (28.80)	
Married/Living with partner	6,106 (67.77)	3,989 (66.12)	2,117 (71.20)	
**Drinking status**				0.42
No	4,946 (51.48)	3,282 (51.06)	1,664 (52.35)	
Yes	4,549 (48.52)	3,058 (48.94)	1,491 (47.65)	
**Smoking status**				< 0.0001
No	4,825 (52.23)	3,360 (54.39)	1,465 (47.77)	
Yes	4,670 (47.77)	2,980 (45.61)	1,690 (52.23)	
**DM**				< 0.0001
No	8,079 (88.78)	5,541 (90.70)	2,538 (84.79)	
Yes	1,416 (11.22)	799 (9.30)	617 (15.21)	
**Hypertension**				< 0.0001
No	5,883 (65.77)	4,194 (70.37)	1,689 (56.23)	
Yes	3,612 (34.23)	2,146 (29.63)	1,466 (43.77)	
**CVD**				< 0.0001
No	8,683 (93.81)	5,876 (94.80)	2,807 (91.74)	
Yes	812 (6.19)	464 (5.20)	348 (8.26)	
**Snore**				< 0.0001
No	4,660 (51.49)	4,592 (75.31)	68 (2.09)	
Yes	4,835 (48.51)	1,748 (24.69)	3,087 (97.91)	
**Stop breathing**				< 0.0001
No	8,253 (87.73)	6,292 (99.41)	1,961 (63.51)	
Yes	1,242 (12.27)	48 (0.59)	1,194 (36.49)	
**Daytime sleepiness**				< 0.0001
No	8,867 (93.70)	6,114 (96.35)	2,753 (88.20)	
Yes	628 (6.30)	226 (3.65)	402 (11.80)	

BRI, Body Roundness Index; BMI, Body Mass Index; PIR, Poverty Income Ratio; GED, general educational development; DM, diabetes mellitus; CVD, Cardiovascular Disease; TG, Triglyceride; TC, Total Cholesterol; HDL, High-Density Lipoprotein Cholesterol.

### 3.2 ROC curve analysis

**Figure 2:**
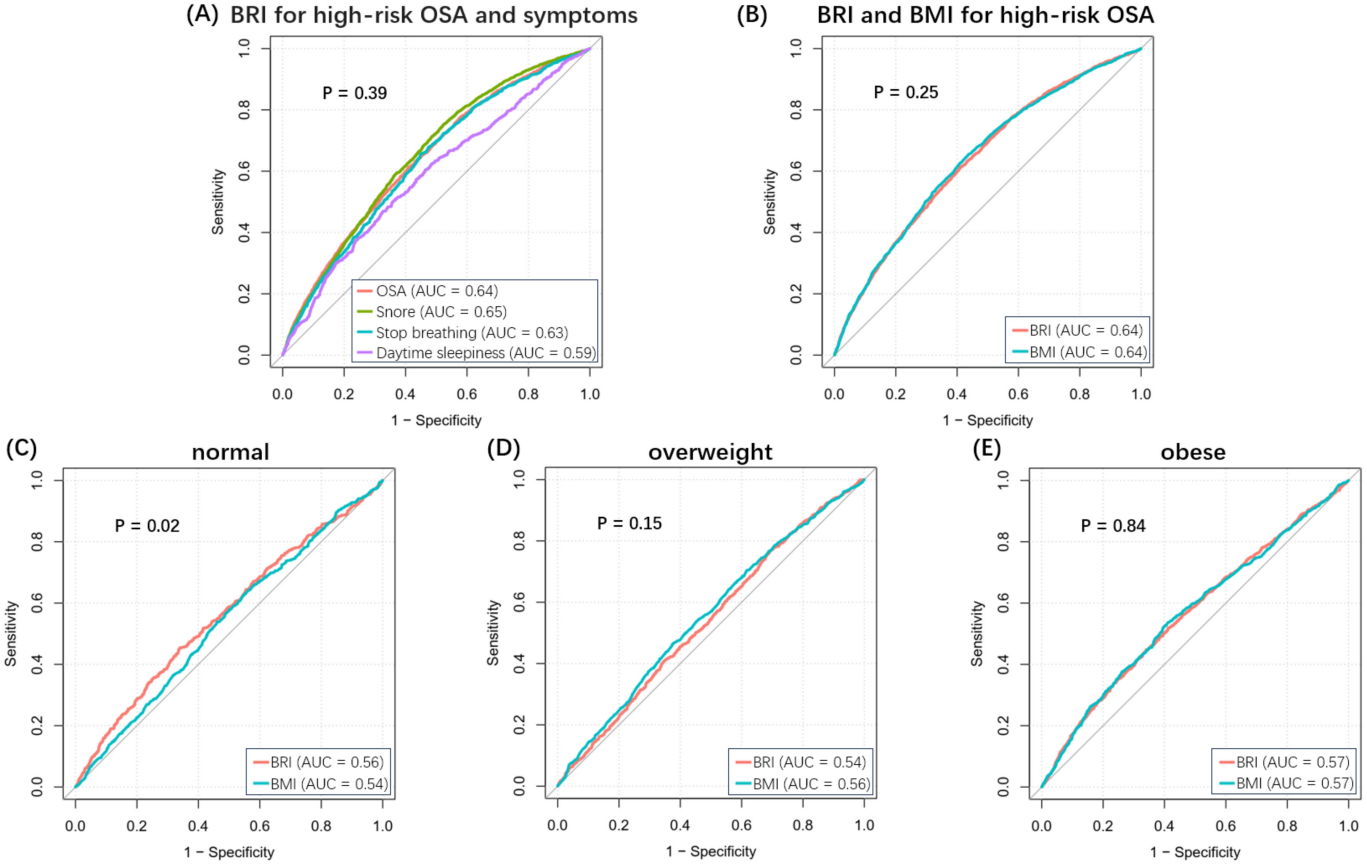
ROC curves for BRI and BMI. **(A)** BRI for high-risk OSA and related symptoms. **(B)** BRI and BMI for high-risk OSA in the overall population. **(C–E)** BRI and BMI for high-risk OSA in normal, overweight, and obese populations.

Figure 2A shows the ROC curves for high-risk for OSA and associated symptoms. The AUC values for the BRI were as follows: 0.64 for OSA, 0.65 for snoring, 0.63 for apnea, and 0.59 for daytime sleepiness. There are no statistically significant comparisons between groups. Similarly, the diagnostic power of the BRI and BMI did not differ significantly in the overall population (Figure 2B). It is noteworthy that the diagnostic power of the BRI was superior to that of BMI in the normal-weight population (*P* < 0.05), although it was not statistically significant in the overweight and obese population (Figures 2C–E). Supplementary Table 4 shows the specific results of the ROC analyses.

### 3.3 Associations of BRI with high-risk for OSA

**Figure 3:**
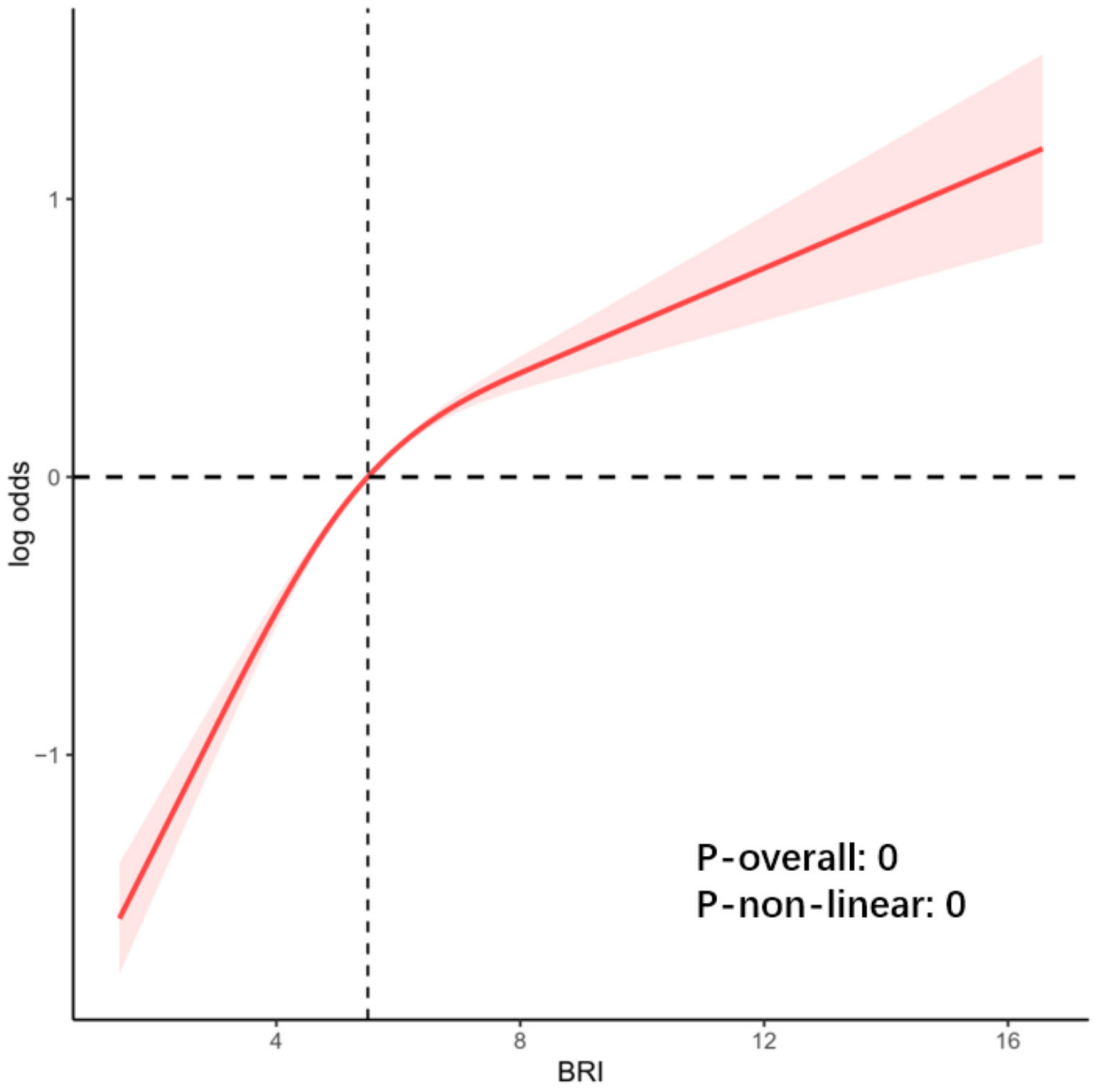
RCS fitting for the association between BRI and OSA risk.

The results of the regression models showed a significant positive correlation between both BRI and BMI and OSA risk (Table 2). However, the correlation between BRI and OSA risk was more potent than BMI. In the crude model, each unit increase in BRI was associated with a 23% increase in the risk of OSA (OR = 1.23; 95% CI: 1.20–1.27; *P* < 0.0001), and each unit increase in BMI was associated with an 8% increase in the risk of OSA (OR = 1.08; 95% CI: 1.06–1.09; *P* < 0.0001). Compared with the lowest quartile (Q1) of BRI, participants in Q4 had a 4.02-fold increased probability of a high risk of OSA (OR = 4.02; 95% CI: 3.30–4.90; *P* < 0.0001). In the fully adjusted model (Model 3), each one-unit increase in BRI was associated with a 19% increase in the risk of OSA (OR = 1.19; 95% CI: 1.16–1.23; *P* < 0.0001), and each one-unit increase in BMI was associated with a corresponding 6% increase in the risk of OSA (OR = 1.06; 95% CI: 1.05–1.08; *P* < 0.0001). In addition, analysis of quartiles of BRI showed a 3.22-fold increase in the probability of having a high risk of OSA at Q4 compared with Q1 (OR = 3.22; 95% CI: 2.57–4.04; *P* < 0.0001). The RCS results demonstrated a non-linear relationship between BRI and OSA risk (Figure 3). The RCS curves for the other symptoms were similar to Figure 3 (Supplementary Figure 1).

**Table 2:** Multivariable logistic regression models for the weighted relationship between BRI and OSA risk.

**OSA**	**OR (95%CI)**, ***P*****-value**			
	**Crude model**	**Model 1**	**Model 2**	**Model 3**
BRI	1.23 (1.20,1.27), < 0.0001	1.24 (1.20,1.28), < 0.0001	1.19 (1.16,1.23), < 0.0001	1.19 (1.16,1.23), < 0.0001
BMI	1.08 (1.06,1.09), < 0.0001	1.08 (1.06,1.09), < 0.0001	1.06 (1.05,1.08), < 0.0001	1.06 (1.05,1.08), < 0.0001
**BRI quartile**				
Q1	Ref	Ref	Ref	Ref
Q2	1.91 (1.60,2.28), < 0.0001	1.80 (1.50,2.15), < 0.0001	1.66 (1.38,2.00), < 0.0001	1.67 (1.39,2.02), < 0.0001
Q3	2.71 (2.18,3.35), < 0.0001	2.53 (2.02,3.16), < 0.0001	2.19 (1.74,2.75), < 0.0001	2.21 (1.75,2.80), < 0.0001
Q4	4.02 (3.30,4.90), < 0.0001	4.02 (3.26,4.95), < 0.0001	3.20 (2.57,3.99), < 0.0001	3.22 (2.57,4.04), < 0.0001
*P* for trend	< 0.0001	< 0.0001	< 0.0001	< 0.0001

Crude model: unadjusted.

Model 1: adjusted for age, sex, marital status, PIR and educational level.

Model 2: adjusted for age, sex, marital status, PIR, educational level, DM, Hypertension, CVD, TG, TC, HDL and smoking status.

Model 3: adjusted for age, sex, race, marital status, PIR, educational level, DM, Hypertension, CVD, TG, TC, HDL, smoking status and drinking status.

OSA, Obstructive Sleep Apnea; BRI, Body Roundness Index; BMI, Body Mass Index.

### 3.4 Subgroup analyses

**Figure 4:**
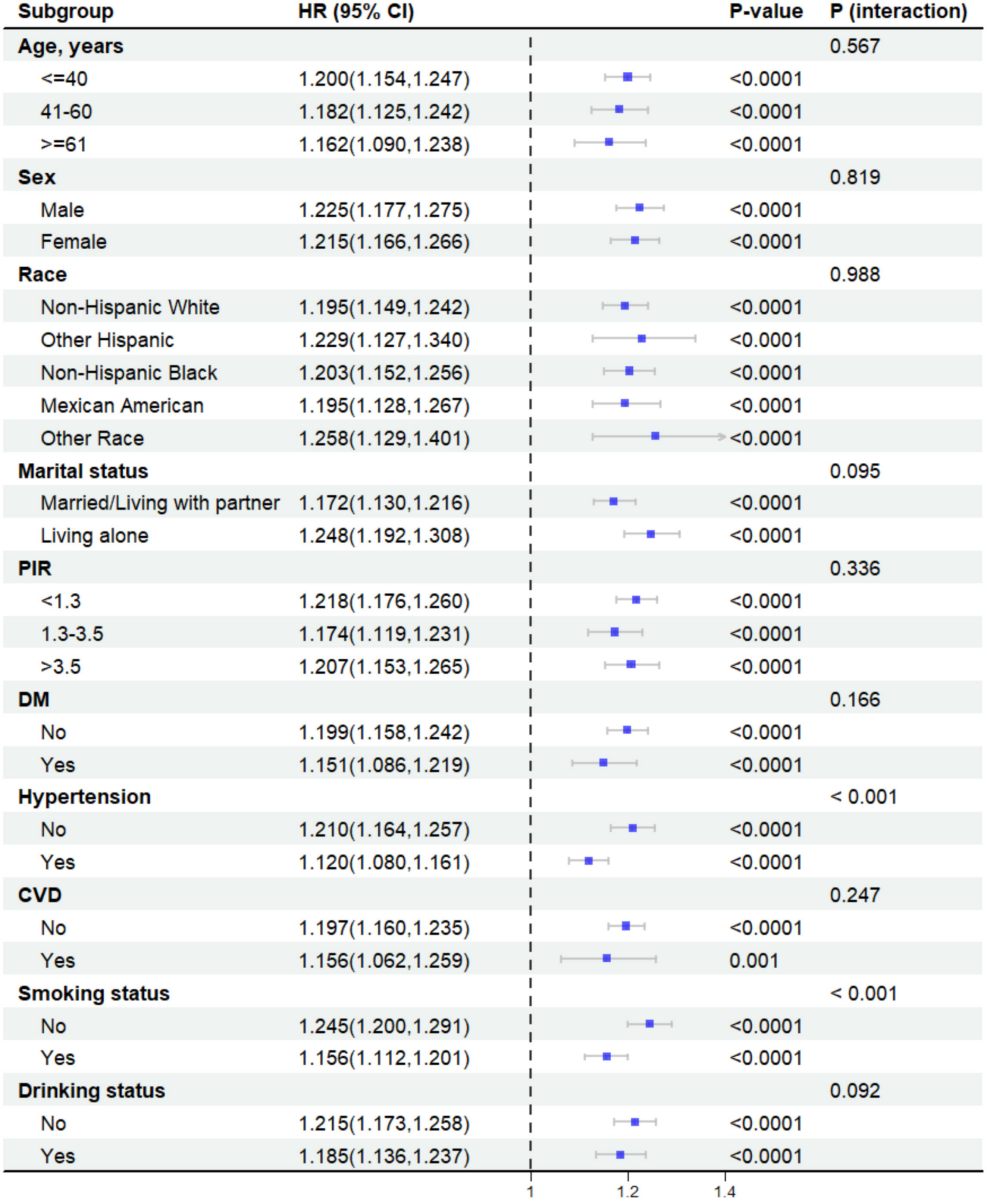
Subgroup analyses. After being adjusted for age, sex, race, marital status, PIR, educational level, DM, Hypertension, CVD, TG, TC, HDL, smoking status and drinking status.

In the weighted subgroup analysis based on model 3, a significant association between higher BRI and high risk of OSA was observed in all subgroups (Figure 4). However, there was a significant interaction between hypertension and smoking status and association (*P* for interaction < 0.001). Specifically, the association between BRI and the risk of OSA was more evident in the subgroups of participants with no hypertension or non-smoking status. No significant interactions were found in other subgroups.

## 4 Discussion

In this large-scale study involving 9,495 representative American adults aged 20 years and older, we found a significant positive association between BRI and the risk of OSA. After adjustment for covariates, the positive association was still observed. The subgroup analyses further confirmed the stability of this correlation. Furthermore, RCS analyses showed a non-linear relationship between BRI levels and OSA risk. The results imply that early monitoring and control of BRI is beneficial in reducing the risk of OSA.

Obesity is significantly associated with the incidence of OSA and influences the severity of OSA (23). It has been shown that in obese patients, it is abdominal visceral fat deposition, rather than subcutaneous fat area, that is associated with OSA (10, 24, 25). Therefore, BMI is limited in its ability to identify people at high risk of OSA because it does not accurately reflect the true distribution of body fat in obese patients. Currently, there are multiple indexes for determining visceral fat accumulation, but their focuses are not the same. The weight-adjusted waist circumference index (WWI) combines weight and waist circumference, focusing on the relationship between abdominal fat accumulation and body weight. Zhang et al.'s cross-sectional study based on 2013–2020 NHANES data found that elevated levels of WWI were associated with an increased risk of OSA (21). However, WWI does not reflect the height factor; thus, BRI is more suitable for comparing the body fat status of individuals of different heights. Lipid accumulation product (LAP) is calculated from triglycerides and waist circumference, and visceral adiposity index (VAI) is calculated from waist circumference, BMI, triglycerides, and high-density lipoproteins. LAP and VAI primarily assess obesity by focusing on metabolic health and fat accumulation. Zhou et al. found that the levels of LAP and VAI were significantly correlated with the risk of OSA and can be used as a predictor of OSA. LAP and VAI levels are also associated with an increased risk of OSA (26). However, since both LAP and VAI require the detection of TG levels during the calculation process, they are not as simple and convenient as BRI in screening high-risk OSA populations. The BRI, as a new obesity assessment index, has the advantage of visualizing body shape and fat distribution.

In this study, we also performed a detailed comparison between BRI and BMI, and the risk of OSA was more sensitive to changes in BRI levels than in BMI levels. This result further confirms the effect of central obesity on OSA and shows the potential value of BRI in identifying OSA risk, indicating that BRI can be used as part of a predictive model for assessing OSA risk. Currently, numerous OSA risk screening questionnaires include BMI, such as the STOP-Bang questionnaire and the Berlin Questionnaire (27, 28). Based on the results of this study, subsequent attempts could be made to use the BRI as a supplement to, or a substitute for, BMI. The ROC analyses showed that, although there was no significant difference between the diagnostic ability of the BRI and BMI in the overall population for the high-risk group for OSA, the BRI performed better in the normal-weight population. Compared with BMI, the stronger recognition ability of BRI also confirms the finding that visceral fat accumulation is associated with OSA risk (25). These findings not only emphasize the importance of weight loss interventions in controlling the risk of OSA but also provide strong support for the use of BRI in identifying people at high risk of OSA.

**Figure 5:**
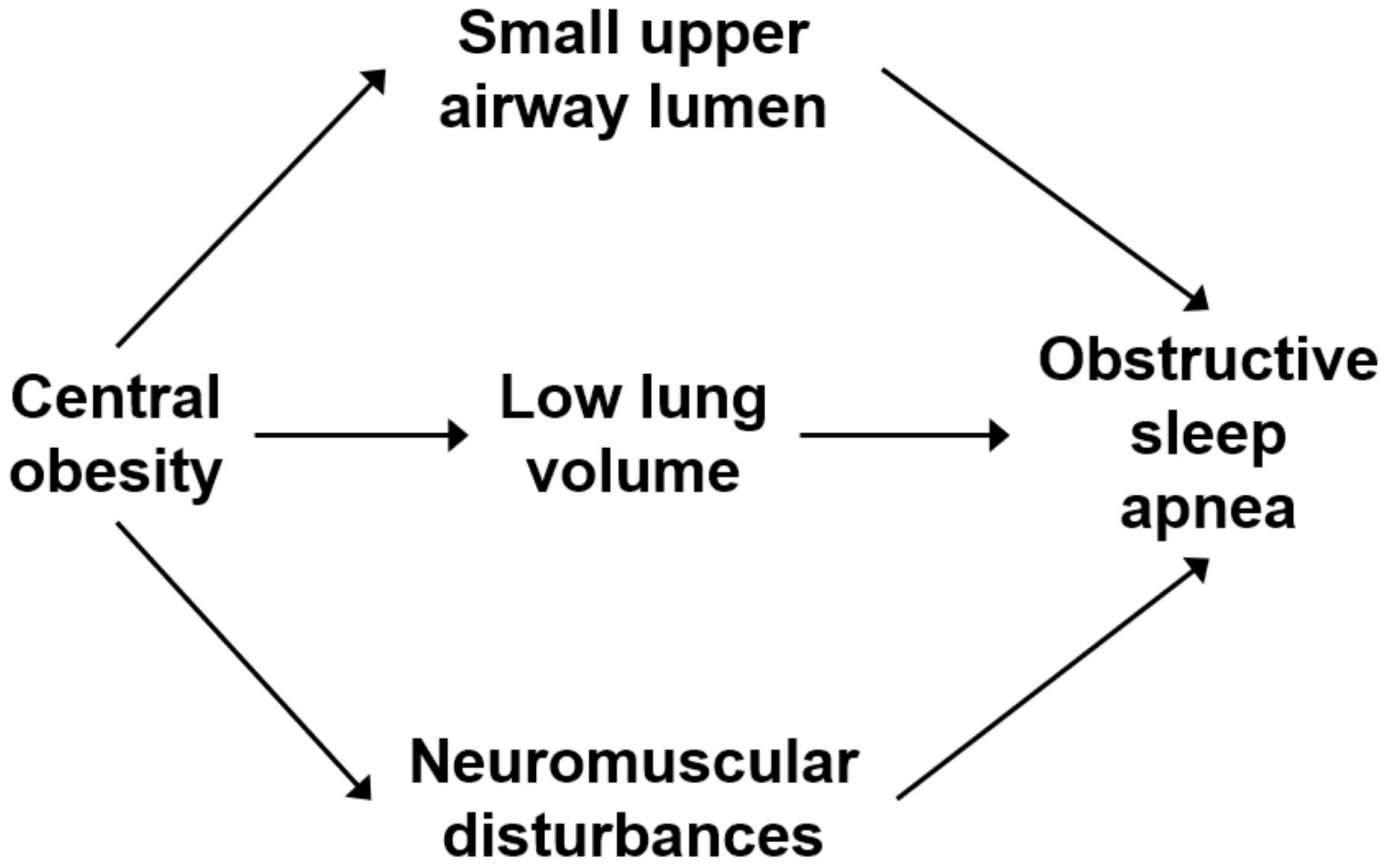
The mechanism between central obesity and OSA.

OSA is characterized by recurrent upper airway obstruction during sleep, mainly due to the disruption of the balance between the negative pressure in the upper airway and the traction force of the upper airway muscles during inspiratory procedures. The mechanism between central obesity, indicated by elevated BRI levels, and OSA is complex, and there are several main explanations (Figure 5). First, central obesity can lead to the accumulation of adipose tissue around the pharyngeal cavity, directly contributing to the narrowing of the upper airway (29). The narrowed upper airway generates larger negative pressure during inspiratory breathing, and the pharyngeal muscle traction on the upper airway is weakened during sleep (30). So, the possibility of upper airway obstruction increases. Second, central obesity leads to a decrease in functional residual capacity by decreasing thoracic compliance on the one hand and by increasing intra-abdominal pressure and pushing the diaphragm cephalad on the other hand. Reduced functional residual capacity decreases the longitudinal traction of the trachea on the pharynx, resulting in a more collapsed upper airway (31, 32). Finally, obesity induces an inflammatory state. Excessive accumulation of visceral adipose tissue produces several inflammatory cytokines such as interleukin-6 (IL-6) and tumor necrosis factor-α (TNF-α) (33). These inflammatory factors increase the likelihood of airway collapse by inhibiting neural control of the upper airway muscles, which cannot provide sufficient dilatory force to maintain airway patency (34).

Subgroup analyses showed a significant interaction effect of hypertension and smoking status on the association (*P* for interaction < 0.001). The association between BRI and OSA risk was more significant in the non-hypertensive population (HR = 1.21 vs. 1.12 in the hypertensive group). It is well known that hypertension is strongly associated with OSA, and epidemiologic surveys have shown that the prevalence of OSA is as high as 30%−40% in hypertensive groups (35). In hypertension and OSA, sympathetic nervous system excitation, oxidative stress, and vascular endothelial dysfunction play important roles (36). The close association between the two may attenuate the independent role of BRI. Secondly, the hypertension-related fluid can narrow the pharyngeal airway by displacing from the legs to the neck during sleep (37). This transfer may cover the direct association of BRI with OSA. In addition, the diagnostic criteria for hypertension in this study included having taken antihypertensive drugs. Several previous studies have explored the effects of antihypertensive drugs on OSA, confirming that diuretics may improve apnea frequency by decreasing fluid levels in patients with OSA and that the angiotensin converting enzyme inhibitor (ACEI) may also decrease apnea frequency and apnea index (38–40). It suggests that antihypertensive drugs may contribute to weakening the effects of BRI. In the smoking status strata, the correlation between BRI and the risk of OSA was similarly observed to be more significant in non-smokers (Hazard Ratio = 1.25 vs. 1.16 in the smoking group), indicating that OSA may be related to smoking. A recent study found that smoking in adults was significantly associated with a higher prevalence of OSA, which is similar to the results of this survey (41). This result may be due to the airway inflammation and damage caused by cigarettes, leading to impaired neuromuscular regulation of the upper airway, increasing its collapsibility and thus partially hiding the impact of changes in the BRI (42).

In this study, we used large-scale data to explore the detailed relationship between BRI and OSA risk. On the one hand, thorough adjustment by confounders improved the credibility of the findings; on the other hand, a comprehensive subgroup analysis was conducted to identify the sensitivity of different populations to changes in BRI levels. Compared to BMI, the advantages of BRI in identifying high-risk OSA populations were also compared from multiple perspectives. However, there are some limitations of this study. First, although the questionnaire information can screen people at high risk of OSA without relying on specialized instruments, there may be data bias caused by memories. Second, due to data limitations, the effect of severe respiratory diseases on OSA could not be considered. Finally, this study was a cross-sectional study, which could not effectively determine causality and a well-designed prospective cohort study is needed for further exploration in the future.

## 5 Conclusion

In summary, this survey confirms that there is a non-linear correlation between BRI and OSA risk, that OSA risk is more sensitive to changes in BRI levels compared to BMI, and that BRI is more effective in identifying those at high risk for OSA within normal-weight population. This suggests that clinical monitoring of BRI can help improve the ability to identify high-risk population for OSA at an early stage.

## Data Availability

Publicly available datasets were analyzed in this study. This data can be found at: https://www.cdc.gov/nchs/nhanes/.
