# Supplementary material for: Relationship between body roundness index and obstructive sleep apnea: a population-based study
Source: Front Nutr. 2025 Mar 26;12:1531006. doi: 10.3389/fnut.2025.1531006 (PMC11980634; doi:10.3389/fnut.2025.1531006)
Supplement: Supplementary file 1 [file Data_Sheet_1.docx]

Supplementary Material

# Supplementary Figures and Tables

## Supplementary Tables

Supplementary Table 1 Weighted univariate logistic regression models for the relationship between BRI and OSA risk

| **Characteristics** | Estimate | Std. Error | t value | Pr(>\|t\|) | OR | 95% CI |
| --- | --- | --- | --- | --- | --- | --- |
| Age (years) | 0.01 | 0 | 5.78 | <0.0001 | 1.01 | 1.01(1.01,1.01) |
| Sex |  |  |  |  |  |  |
| Female | ref | ref | ref | ref | ref | ref |
| Male | 0.39 | 0.07 | 5.86 | <0.0001 | 1.47 | 1.47(1.29,1.68) |
| Race |  |  |  |  |  |  |
| Non-Hispanic Black | ref | ref | ref | ref | ref | ref |
| Mexican American | -0.08 | 0.08 | -0.96 | 0.34 | 0.92 | 0.92(0.78,1.09) |
| Other Hispanic | -0.08 | 0.09 | -0.93 | 0.36 | 0.92 | 0.92(0.77,1.10) |
| Other Race | -0.13 | 0.1 | -1.25 | 0.22 | 0.88 | 0.88(0.72,1.08) |
| Non-Hispanic White | -0.09 | 0.07 | -1.39 | 0.17 | 0.91 | 0.91(0.80,1.04) |
| Marital status |  |  |  |  |  |  |
| Living alone | ref | ref | ref | ref | ref | ref |
| Married/Living with partner | 0.24 | 0.06 | 3.7 | <0.001 | 1.27 | 1.27(1.11,1.44) |
| PIR | -0.04 | 0.02 | -1.85 | 0.07 | 0.96 | 0.96(0.92,1.00) |
| Educational level |  |  |  |  |  |  |
| Above high school | ref | ref | ref | ref | ref | ref |
| High school or GED | 0.18 | 0.07 | 2.51 | 0.01 | 1.2 | 1.20(1.04,1.39) |
| Less than high school | 0.18 | 0.08 | 2.13 | 0.04 | 1.2 | 1.20(1.01,1.42) |
| BRI | 0.21 | 0.02 | 13.89 | <0.0001 | 1.23 | 1.23(1.20,1.27) |
| BMI (kg/m^2^) | 0.07 | 0.01 | 13 | <0.0001 | 1.08 | 1.08(1.06,1.09) |
| TG (mmol/L) | 0.15 | 0.02 | 6.49 | <0.0001 | 1.16 | 1.16(1.11,1.21) |
| TC (mmol/L) | 0.05 | 0.03 | 1.71 | 0.09 | 1.05 | 1.05(0.99,1.11) |
| HDL (mmol/L) | -0.79 | 0.07 | -11.34 | <0.0001 | 0.45 | 0.45(0.40,0.52) |
| DM |  |  |  |  |  |  |
| No | ref | ref | ref | ref | ref | ref |
| Yes | 0.56 | 0.09 | 6.35 | <0.0001 | 1.75 | 1.75(1.47,2.09) |
| Hypertension |  |  |  |  |  |  |
| No | ref | ref | ref | ref | ref | ref |
| Yes | 0.61 | 0.06 | 9.91 | <0.0001 | 1.85 | 1.85(1.63,2.09) |
| CVD |  |  |  |  |  |  |
| No | ref | ref | ref | ref | ref | ref |
| Yes | 0.5 | 0.09 | 5.29 | <0.0001 | 1.64 | 1.64(1.36,1.98) |
| Smoking status |  |  |  |  |  |  |
| No | ref | ref | ref | ref | ref | ref |
| Yes | 0.27 | 0.06 | 4.45 | <0.0001 | 1.3 | 1.30(1.16,1.47) |
| Drinking status |  |  |  |  |  |  |
| No | ref | ref | ref | ref | ref | ref |
| Yes | -0.05 | 0.06 | -0.81 | 0.42 | 0.95 | 0.95(0.84,1.08) |
| Snore |  |  |  |  |  |  |
| No | ref | ref | ref | ref | ref | ref |
| Yes | 4.96 | 0.16 | 31.07 | <0.0001 | 143.09 | 143.09(103.95,196.97) |
| Stop breathing |  |  |  |  |  |  |
| No | ref | ref | ref | ref | ref | ref |
| Yes | 4.56 | 0.2 | 22.88 | <0.0001 | 96 | 96.00(64.42,143.07) |
| Daytime sleepiness |  |  |  |  |  |  |
| No | ref | ref | ref | ref | ref | ref |
| Yes | 1.26 | 0.13 | 9.39 | <0.0001 | 3.53 | 3.53(2.70,4.62) |
| BRI quartile |  |  |  |  |  |  |
| Q1 | ref | ref | ref | ref | ref | ref |
| Q2 | 0.65 | 0.09 | 7.24 | <0.0001 | 1.91 | 1.91(1.60,2.28) |
| Q3 | 1 | 0.11 | 9.3 | <0.0001 | 2.71 | 2.71(2.18,3.35) |
| Q4 | 1.39 | 0.1 | 14.11 | <0.0001 | 4.02 | 4.02(3.30,4.90) |

BRI, Body Roundness Index; BMI: Body Mass Index; PIR, Poverty Income Ratio; GED, general educational development; DM, diabetes mellitus; CVD, Cardiovascular Disease; TG, Triglyceride; TC, Total Cholesterol; HDL, High-Density Lipoprotein Cholesterol.

Supplementary Table 2 Generalized Variance Inflation Factor results

| Characteristics | GVIF | DF | GVIF^(1/(2*Df)) |
| --- | --- | --- | --- |
| BRI | 1.30190428 | 1 | 1.1410102 |
| Age (years) | 1.61091664 | 1 | 1.26921891 |
| Sex | 1.24758508 | 1 | 1.11695348 |
| Race | 1.3495312 | 4 | 1.0381805 |
| Marital status | 1.09503859 | 1 | 1.04644091 |
| PIR | 1.30961795 | 1 | 1.1443854 |
| Educational level | 1.32291151 | 2 | 1.07246394 |
| DM | 1.20827846 | 1 | 1.0992172 |
| Hypertension | 1.34656072 | 1 | 1.16041403 |
| CVD | 1.19034112 | 1 | 1.09102755 |
| TG (mmol/L) | 1.54954076 | 1 | 1.24480551 |
| TC (mmol/L) | 1.34261877 | 1 | 1.15871427 |
| HDL (mmol/L) | 1.5914794 | 1 | 1.26153851 |
| Smoking status | 1.12452302 | 1 | 1.0604353 |
| Drinking status | 1.15280344 | 1 | 1.07368685 |

GVIF = Generalized Variance Inflation Factor; DF = Degrees of Freedom; BRI, Body Roundness Index; PIR, Poverty Income Ratio; DM, diabetes mellitus; CVD, Cardiovascular Disease; TG, Triglyceride; TC, Total Cholesterol; HDL, High-Density Lipoprotein Cholesterol.

Supplementary Table 3 Weighted baseline characteristics according to quartiles of body roundness index

| **Characteristics** | **Total**  **(N = 9495)** | **Quartiles of BRI** | | | | ***P*-value** |
| --- | --- | --- | --- | --- | --- | --- |
|  |  | **Q1**  **(<3.73)** | **Q2**  **(3.73-4.98)** | **Q3**  **(4.98-6.50)** | **Q4**  **(>=6.50)** |  |
| **Continuous variables, mean (SD)** |  |  |  |  |  |  |
| Age (years) | 45.18±0.39 | 37.79±0.49 | 46.02±0.45 | 48.83±0.54 | 49.33±0.51 | < 0.0001 |
| PIR | 3.30±0.04 | 3.30±0.05 | 3.44±0.05 | 3.33±0.06 | 3.12±0.06 | < 0.001 |
| TG (mmol/L) | 1.71±0.03 | 1.15±0.02 | 1.72±0.05 | 2.04±0.05 | 2.05±0.04 | < 0.0001 |
| TC (mmol/L) | 5.03±0.02 | 4.76±0.03 | 5.17±0.03 | 5.21±0.03 | 5.01±0.04 | < 0.0001 |
| HDL (mmol/L) | 1.41±0.01 | 1.61±0.01 | 1.44±0.01 | 1.31±0.01 | 1.25±0.01 | < 0.0001 |
| BMI (kg/m^2^) | 28.90±0.13 | 22.45±0.07 | 26.46±0.07 | 30.26±0.07 | 37.79±0.15 | < 0.0001 |
| **Categorical variables, n (%)** |  |  |  |  |  |  |
| Sex |  |  |  |  |  | < 0.0001 |
| Female | 4319(48.05) | 1101(51.29) | 943(42.00) | 965(42.00) | 1310(56.81) |  |
| Male | 5176(51.95) | 1272(48.71) | 1429(58.00) | 1410(58.00) | 1065(43.19) |  |
| Educational level |  |  |  |  |  | < 0.0001 |
| Above high school | 5560(65.91) | 1548(71.46) | 1412(68.35) | 1285(60.38) | 1315(62.32) |  |
| High school or GED | 2173(22.99) | 485(19.54) | 523(20.79) | 578(26.55) | 587(25.82) |  |
| Less than high school | 1762(11.10) | 340(9.00) | 437(10.85) | 512(13.07) | 473(11.86) |  |
| Race |  |  |  |  |  | < 0.0001 |
| Non-Hispanic Black | 1856(9.08) | 524(9.80) | 414(7.96) | 397(7.84) | 521(10.69) |  |
| Mexican American | 1519(7.80) | 198(4.18) | 389(8.02) | 502(10.68) | 430(8.93) |  |
| Other Hispanic | 845(4.78) | 150(3.66) | 208(5.14) | 258(5.78) | 229(4.70) |  |
| Other Race | 916(6.45) | 330(7.97) | 275(7.01) | 187(6.13) | 124(4.39) |  |
| Non-Hispanic White | 4359(71.89) | 1171(74.39) | 1086(71.87) | 1031(69.56) | 1071(71.29) |  |
| Marital status |  |  |  |  |  | < 0.0001 |
| Living alone | 3389(32.23) | 1024(38.87) | 739(28.37) | 754(28.55) | 872(32.24) |  |
| Married/Living with partner | 6106(67.77) | 1349(61.13) | 1633(71.63) | 1621(71.45) | 1503(67.76) |  |
| Drinking status |  |  |  |  |  | 0.01 |
| No | 4946(51.48) | 1172(47.86) | 1255(52.65) | 1279(53.38) | 1240(52.56) |  |
| Yes | 4549(48.52) | 1201(52.14) | 1117(47.35) | 1096(46.62) | 1135(47.44) |  |
| Smoking status |  |  |  |  |  | 0.001 |
| No | 4825(52.23) | 1272(55.90) | 1248(53.75) | 1132(49.14) | 1173(49.40) |  |
| Yes | 4670(47.77) | 1101(44.10) | 1124(46.25) | 1243(50.86) | 1202(50.60) |  |
| DM |  |  |  |  |  | < 0.0001 |
| No | 8079(88.78) | 2292(98.08) | 2141(94.12) | 1966(86.80) | 1680(74.05) |  |
| Yes | 1416(11.22) | 81(1.92) | 231(5.88) | 409(13.20) | 695(25.95) |  |
| Hypertension |  |  |  |  |  | < 0.0001 |
| No | 5883(65.77) | 1962(84.74) | 1559(69.42) | 1330(59.92) | 1032(45.36) |  |
| Yes | 3612(34.23) | 411(15.26) | 813(30.58) | 1045(40.08) | 1343(54.64) |  |
| CVD |  |  |  |  |  | < 0.0001 |
| No | 8683(93.81) | 2292(98.00) | 2225(95.39) | 2123(92.61) | 2043(88.37) |  |
| Yes | 812(6.19) | 81(2.00) | 147(4.61) | 252(7.39) | 332(11.63) |  |
| OSA |  |  |  |  |  | < 0.0001 |
| Low-risk | 6340(67.46) | 1927(81.78) | 1660(70.15) | 1521(62.40) | 1232(52.77) |  |
| High-risk | 3155(32.54) | 446(18.22) | 712(29.85) | 854(37.60) | 1143(47.23) |  |
| Snore |  |  |  |  |  | < 0.0001 |
| No | 4660(51.49) | 1634(70.73) | 1211(53.07) | 1004(42.77) | 811(35.86) |  |
| Yes | 4835(48.51) | 739(29.27) | 1161(46.93) | 1371(57.23) | 1564(64.14) |  |
| Stop breathing |  |  |  |  |  | < 0.0001 |
| No | 8253(87.73) | 2218(94.37) | 2125(90.17) | 2010(83.71) | 1900(81.30) |  |
| Yes | 1242(12.27) | 155(5.63) | 247(9.83) | 365(16.29) | 475(18.70) |  |
| Daytime sleepiness |  |  |  |  |  | < 0.0001 |
| No | 8867(93.70) | 2249(94.54) | 2262(95.53) | 2217(94.08) | 2139(90.36) |  |
| Yes | 628(6.30) | 124(5.46) | 110(4.47) | 158(5.92) | 236(9.64) |  |

BRI, Body Roundness Index; BMI: Body Mass Index; PIR, Poverty Income Ratio; GED, general educational development; DM, diabetes mellitus; CVD, Cardiovascular Disease; TG, Triglyceride; TC, Total Cholesterol; HDL, High-Density Lipoprotein Cholesterol.

Supplementary Table 4 Diagnostic Statistics of BRI and BMI in Different Groups

| Characteristics | AUC (95%CI) | Threshold | Sensitivity | Specificity | Youden index | *P*-value |
| --- | --- | --- | --- | --- | --- | --- |
| All participants |  |  |  |  |  | 0.25 |
| BRI | 0.64 (0.63,0.65) | 5.08 | 0.61 | 0.59 | 0.20 |  |
| BMI | 0.64 (0.63,0.66) | 27.81 | 0.65 | 0.56 | 0.22 |  |
| BMI group |  |  |  |  |  |  |
| Normal |  |  |  |  |  | 0.02 |
| BRI | 0.56 (0.54,0.59) | 3.46 | 0.45 | 0.66 | 0.11 |  |
| BMI | 0.54 (0.51,0.57) | 22.16 | 0.63 | 0.45 | 0.08 |  |
| Overweight |  |  |  |  |  | 0.16 |
| BRI | 0.54 (0.52,0.56) | 4.26 | 0.77 | 0.31 | 0.08 |  |
| BMI | 0.56 (0.54,0.58) | 27.81 | 0.47 | 0.62 | 0.09 |  |
| Obese |  |  |  |  |  | 0.84 |
| BRI | 0.57 (0.55,0.59) | 7.60 | 0.45 | 0.66 | 0.11 |  |
| BMI | 0.57 (0.55,0.59) | 34.69 | 0.54 | 0.59 | 0.13 |  |

BRI, Body Roundness Index; BMI: Body Mass Index.

## Supplementary Figures

Supplementary Figure 1.

**
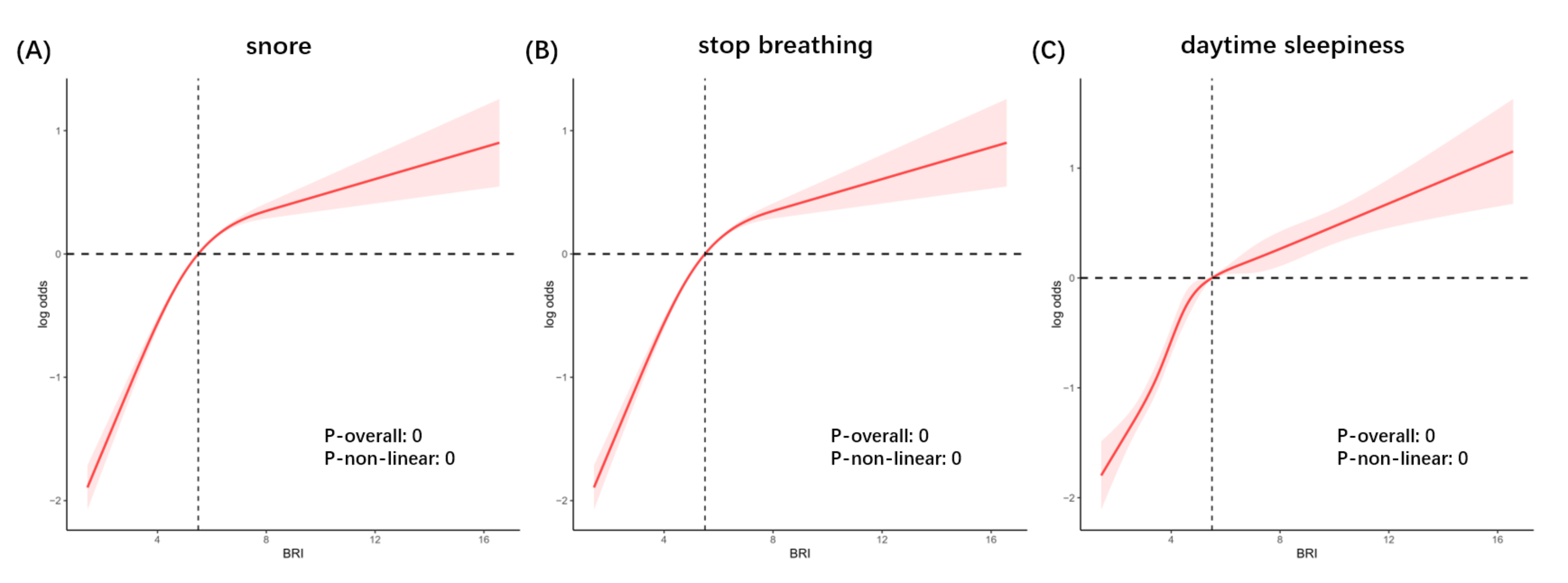
**

RCS fitting for the association between BRI and snore, stop breathing, and daytime sleepiness. BRI, Body Roundness Index.
